# Evaluating Information Quality of Revised Patient Education Information on Colonoscopy: It Is New But Is It Improved?

**DOI:** 10.2196/11938

**Published:** 2019-02-20

**Authors:** Matthew Tyler Bernstein, James Kong, Vaelan Sriranjan, Sofia Reisdorf, Gayle Restall, John Roger Walker, Harminder Singh

**Affiliations:** 1 Department of Clinical Health Psychology University of Manitoba Winnipeg, MB Canada; 2 Inflammatory Bowel Disease Clinical and Research Centre University of Manitoba Winnipeg, MB Canada; 3 Department of Occupational Therapy University of Manitoba Winnipeg, MB Canada; 4 Section of Gastroenterology Department of Internal Medicine University of Manitoba Winnipeg, MB Canada

**Keywords:** colonoscopy, evaluation methodology, information science, information dissemination, information literacy

## Abstract

**Background:**

Previous research indicates that patients and their families have many questions about colonoscopy that are not fully answered by existing resources. We developed revised forms on colonoscopy bowel preparation and on the procedure itself.

**Objective:**

As the goal of the revised materials is to have improved information relative to currently available information, we were interested in how revised information compared with what is currently available in terms of information quality and patient preference.

**Methods:**

Participants were asked to review one at a time the Revised and Current versions of *Colonoscopy bowel preparation instructions* (study 1) and *About Colonoscopy* (study 2). The order of administration of the Revised and Current versions was randomly counterbalanced to assess order effects*.* Respondents rated each form along the following dimensions: amount, clarity, trustworthiness, readability and understandability, how new or familiar the information was, and reassurance. Participants were asked which form they preferred and 4 questions about why they preferred it. Open-ended questions asked participants to describe likes and dislikes of the forms and suggestions for improvement.

**Results:**

The study 1 and study 2 samples were similar. Overall, in study 1, 62.4% preferred the Revised form, 28.1% preferred the Current form, and 6.7% were not sure. Overall, in study 2, 50.5% preferred the Revised form, 31.1% preferred the Current form, and 18.4% were not sure. Almost 75% of those in study 1 who received the Revised form first, preferred it, compared with less than half of those who received it first in study 2. In study 1, 75% of those without previous colonoscopy experience preferred the Revised form, compared with more than half of those who had previously undergone a colonoscopy. The study 1 logistic regression analysis demonstrated that participants were more likely to prefer the Revised form if they had viewed it first and had no previous experience with colonoscopy. In study 2, none of the variables assessed were associated with a preference for the Revised form. In comparing the 2 forms head-to-head, participants who preferred the Revised form in study 1 rated it as clearer compared with those who preferred the Current form. Finally, many participants who preferred the Revised form indicated in the open-ended questions that they liked it because it had more information than the Current form and that it had good visual information.

**Conclusions:**

This study is one of the first to evaluate 2 different patient education resources in a head-to-head comparison using the same participants in a within-subjects design. This approach was useful in comparing revised educational information with current resources. Moving forward, this knowledge translation approach of a head-to-head comparison of 2 different information sources could be taken to develop and refine information sources on other health issues.

## Introduction

### Background

Colorectal cancer is the second most common cancer diagnosis and cause of cancer-related deaths among men and the third most common cancer diagnosis and cause of cancer-related deaths among women [1]. Many of these deaths can be prevented by screening for colorectal cancer and colorectal polyps. Colonoscopy is an essential initial test or follow-up for other positive tests for colorectal cancer screening. Colonoscopy is widely used for assessing and removing the polyps that can lead to colorectal cancer and early detection of colorectal cancers as well for evaluating a range of different gastrointestinal symptoms. To ensure that the colonoscopy procedure is successful (ie, accurate viewing of the colon), an individual must undergo a demanding preparation, which involves cleansing of the colon of residual materials. Inadequate cleansing of the colon can lead to missed detection and diagnosis of colorectal pathologies such as colorectal cancer and polyps. However, 10% to 20% of colonoscopies fail because of poor preparation [2]. Poor preparation can also lead to increased duration and repetition of the colonoscopy [3], which, in addition to recipient inconvenience and worse health care outcomes, leads to increased costs [4]. One way to safeguard against poor preparation is by educating patients about best preparation practices with information that is clear and engaging [5-7]. Recent systematic reviews found that patient education interventions improve the quality of bowel preparation [7] and reduce anxiety about the procedure [8]. Clinical practices use a range of different materials to inform patients about colonoscopy preparation [9], but there has been limited evaluation of the quality of the materials used and limited efforts to improve the quality of these materials.

A recent study by our group [10] explored the information needs and preferences of patients undergoing colonoscopy. The results of this study indicated that some patients feel inadequately informed about the colonoscopy procedure, and those receiving their first colonoscopy felt less informed than those who had received one in the past. Most participants (90%) also indicated that speaking with a family doctor about a colonoscopy would have been helpful or very helpful; however, only 20% to 26% reported having received the right amount of colonoscopy information from their family doctor.

Recent studies have compared different approaches of providing information to patients about colonoscopy. A randomized controlled trial (RCT) compared standard written instructions with written instructions plus a video that provided visual instructions about the preparation process as well as figures depicting optimal and poor bowel preparation [11]. Patients in the video condition had better ratings of bowel preparation than those in the standard instructions condition, but there was no difference in satisfaction with the procedure. Another RCT compared patients who were having a first-time screening colonoscopy after reviewing the American Gastroenterological Association colonoscopy educational pamphlet in addition to the standard pamphlet, compared with those who had standard written instructions only [12]. Those in the augmented education group reported lower levels of anxiety, had reduced sedative use during the procedure, and better preparation as rated by the endoscopist [12]. These studies suggest the importance of providing patients with high-quality information before the colonoscopy procedure. Kurlander et al [7] conducted a systematic review of patient education interventions to improve colonoscopy preparation. A total of 7 full-text studies were included in the final analysis. These studies took place in the United States, China, Korea, and Taiwan. In each study, participants were randomized to receive an augmented educational intervention, compared with the educational materials used in usual care. The augmented interventions involved including additional written material (3 studies), videos (2 studies), telephone calls the day before the colonoscopy (1 study), and in-person education by physicians (1 study). In 6 of the 7 studies, there was a significant improvement in bowel preparation scores, compared with usual care [7]. These findings are also supported in a recent meta-analysis that included 8 RCTs [13]. People who received enhanced instructions (ie, regular written and/or verbal instruction plus improved written materials, visual aids, smartphone apps, or additional instruction over the telephone) showed significantly better bowel preparation quality than those receiving standard instructions. Furthermore, individuals who received enhanced instructions were more willing to repeat the procedure than those who did not receive enhanced instructions [13].

Prior studies have often not asked participants about their information needs and/or their assessment of the quality of the provided information and have rather focused on measures such as bowel cleansing on colonoscopy. More importantly, no studies have compared the quality of different augmented interventions or enhanced instructions and hence, it is impossible for guidelines to recommend a standardized or preferred approach [7].

### Information Quality

A considerable amount of research in the social psychology area has used the same participants to evaluate characteristics of two or more different targets (eg, photos or information about persons who may be seen during social contacts). On the other hand, few studies have used this comparison methodology in evaluating the information quality of two (or more) patient information resources. Arazy et al [14] have developed an approach to information quality focused on heuristic principles as a multidimensional construct including dimensions such as accuracy, completeness, objectivity, and representation. Recently, Fidler and Lavbic [15] enrolled university students in an online survey in which they were asked to evaluate 3 selected and rewritten papers from Wikipedia, presented in a random order, on the following dimensions: accuracy, completeness, objectivity, and representation. They found that Wikipedia papers can be enhanced for students by using more socially relevant wording [15]. Yaari et al [16] used a similar design. With a sample of university students, they compared the responses of 1 group of participants to 2 different information sources (using a within-subjects design). The advantage of this methodology is that using the same participants allows for clearer judgments as to whether the characteristics of 1 resource were evaluated more positively than the other.

### Order Effects

In a seminal paper by Murdock [17] on serial order effects in short-term memory, he found there to be a U-shaped (ie, nonlinear) serial position curve regarding recall. This U-shaped curve represents better memory for stimuli presented first (primacy effects) and stimuli presented last (recency effects) and worse memory for stimuli presented in between. Furthermore, there has been a lot of research suggesting that individuals prefer to recall information from memory in forward serial order even when it is not required by the task at hand [18-20]. Although these models have mostly focused on numbers, letters, and words, very little research has been conducted on the order effects of larger quantities of information. Due to this limitation in previous research, we decided to evaluate order effects by using random assignment to counterbalance the order of presentation of the materials being evaluated.

This research builds on the existing research in the evaluation of patient-oriented educational information by having the same individuals compare 2 sources of information. This project involved 2 studies with the purpose of determining individuals’ opinions of the characteristics of information about 2 aspects of colonoscopy: Study 1 evaluated *Colonoscopy bowel preparation instructions* that included time of day to take the bowel preparation, food and drink restrictions, and type of bowel movements to expect. Study 2 evaluated educational material called *About Colonoscopy* that included reasons and risks for having a colonoscopy as well as what happens on the day of the procedure. As the goal of the revised materials is to have improved information relative to currently available information, we were interested in how the revised information can be compared with the currently available information in terms of information quality and patient preference. The currently available material was developed locally, 1 year before our group developed revised materials; revised materials were developed based on feedback from patients and health care providers. Feedback was gathered after using the current materials and then used to improve the revised materials. Patient and provider advisory groups allowed for feedback in developing the revised materials.

## Methods

### Revised Form Development

In 2017, our research team focused on a project titled *Optimizing colonoscopy procedures and reducing unnecessary and over use* and developed revised educational resources for patients referred for colonoscopy. The materials went beyond simply explaining the preparation instructions, and instead, used visual aids and information using clear language with less medical jargon, short sentences, and brief paragraphs with the goal of making the information clearer to the average reader [21,22]. The reader may access these and other educational materials developed by our research team (including videos) at mycolonoscopy website [23]. The written materials have Creative Commons licenses, so they may be used in other settings.

### Readability

In developing patient education materials, developers strive to have content that can be understood by patients who are comfortable reviewing materials at lower reading levels. Studies of material available on the Web [24] indicate a wide range of reading levels in educational materials on the Web—with many materials being so high in reading level that they are difficult for patients to understand. Keeping the reading level of materials at a comfortable level can be challenging in the health area because of the medical terminology used to describe health concerns. Accurate readability calculators are now easily available on the Web. They consider factors such as the length of the words in a passage and length of sentences and paragraphs. We used the version available at [25] to calculate the readability score of the Revised and Current versions of the materials evaluated in Study 1 and Study 2 (ie, the Simple Measure of Gobbledygook [SMOG] Index) [25].

### Participants

Patients were recruited from the waiting room of gastroenterology and urology clinics at the largest hospital in the province of Manitoba, located in Winnipeg’s inner city. The patients were seen in this setting for consultation around a wide variety of gastrointestinal and urological problems. A research assistant approached patients and those accompanying them in the waiting area and invited them (patients and accompanying adults) to complete a survey evaluating 2 sets of information materials. The information materials reviewed by participants in this study were in paper format. The mycolonoscopy.ca website that contains the Revised information allows for downloading and printing of the information materials in addition to viewing them on a Web browser.

### Measurement

Respondents in study 1 were asked to review one at a time the Revised and Current versions of the *Colonoscopy bowel preparation instructions* (order of administration was randomly counterbalanced) and to rate each along the following dimensions: amount of information, clarity, trustworthiness, readability and understandability, how new or familiar the information was (very familiar to very new), and reassurance (very worried to very reassured). These dimensions were rated using 5-point Likert-type scales. Open-ended questions included likes and dislikes about the information form and suggestions for improvement. After participants viewed both forms and responded to these questions, they were asked “Which form do you think would be most helpful for people who are considering having a colonoscopy?” Afterwards, they were asked 4 comparison questions along similar dimensions to those described above (ie, clarity, trustworthiness, readability and understandability, and reassurance). Finally, they were asked an open-ended question about why their preferred form is better than the other form. Participants were also asked some background questions including age, sex, primary language spoken, education, history of gastroenterology visits, and history of a colonoscopy. Participants in study 2 took a similar approach for evaluating the revised and current resources titled *About Colonoscopy*. Survey questions used in this study are shown in Multimedia Appendix 1. Multimedia Appendices 2-5 contain the educational material (Current and Revised forms) on the 2 topics. This study was approved by the University of Manitoba Health Research Ethics Board.

### Statistical Methods

The Web-based calculator available was used to calculate the SMOG readability score for the different forms [25]. Briefly, the SMOG formula counts the number of words with 3 or more syllables from a sample of at least 30 sentences and then takes the square root and adds 3 to obtain the readability score.

IBM SPSS statistics version 24.0 was used to conduct the data analysis. Descriptive statistics (including means and proportions) were used to summarize sociodemographic information and the responses to questions about information form ratings and preferences. CIs were reported, as they are typically used in survey research, and they allow for convenient comparisons within and across different survey questions and groups of respondents. CIs have been recommended rather than pairwise significance tests for this type of comparison because they help the reader to understand the magnitude of differences rather than simply concluding whether or not a difference is statistically significant [26,27].

Logistic regression was used to examine the predictors of preference for the Revised form. The following predictors were used: order, previous colonoscopy, gender, age, education, and language most often spoken at home. A median-split approach was used to transform age and education into dichotomous variables.

Pearson correlations of the variables used to evaluate the 2 forms were calculated to assess whether they assessed different concepts or if some variables were redundant and could be deleted in future surveys. The open-ended questions were analyzed using a descriptive content analysis approach [28]. Authors MTB and GR coded these responses and organized codes into categories.

## Results

As can be seen in Table 1, overall, the samples in study 1 and study 2 were similar. More than half of each sample was female, and they had about 2 years of education after high school. Most of each sample had previously seen a gastroenterologist and previously had a colonoscopy. One noteworthy difference was that the mean age was about 10 years lower in study 1 than in study 2. The study 1 response rate was 78.8% (178/226), compared with a study 2 response rate of 78.5% (219/279). Nearly all the participants in both studies completed the survey if they had started it (96% completion rate in study 1 and 94% in study 2). The Revised forms in both studies yielded SMOG indexes equal to a Grade 8 reading level. The Current form yielded a SMOG index=Grade 7 reading level in Study 1 and a SMOG index=Grade 10 in Study 2.

Overall, in study 1, 62.4% preferred the Revised form, 28.1% preferred the Current form, and 6.7% were not sure. Overall, in study 2, 50.5% preferred the Revised form, 31.1% preferred the Current form, and 18.4% were not sure. Table 2 displays the results for participants’ preferred form based on the order of presentation. Interestingly, almost three-quarters of participants in study 1 who received the Revised form first, preferred it, compared with less than half of those who received it first in study 2. Furthermore, in Study 1, three-quarters of those without previous colonoscopy experience preferred the Revised form, compared with more than half of those who had previously undergone a colonoscopy, whereas in study 2, close to 50% preferred the Revised form irrespective of whether they had a previous colonoscopy or not (Table 3).

**Table 1:** Sociodemographic characteristics of respondents with each order of presentation.

Characteristics		Study 1		Study 2	
		Revised form first (N=86)	Current form first (N=92)	Revised form first (N=103)	Current form first (N=103)
Age (years), mean (95% CI)		42.0 (38.7-45.3)	46.6 (43.4-49.8)	55.2 (52.3-58.1)	55.0 (52.0-8.0)
Female proportion, n (%); 95% CI for %		54 (63); 52-73	61 (66); 56-76	53 (51.4); 41-61	63 (61.2); 51-71
English main language proportion, n (%); 95% CI for %		75 (87); 78-93	87 (94); 88-98	95 (92.2); 85-97	97 (94.2); 88-98
Mean years of education, mean (95% CI)		14.9 (14.2-15.6)	15.2 (14.4-16.0)	14.6 (14.1-15.2)	15.0 (14.3-15.7)
Seen gastroenterologist before? (% yes), n (%); 95% CI for %		61 (71); 60-80	61 (66); 56-76	66 (64.1); 54-73	69 (66.9); 57-76
Colonoscopy before? (% yes), n (%); 95% CI for %		62 (72); 61-81	59 (64); 54-74	67 (65.0); 55-74	66 (64.1); 54-73
**Reason for visit, n (%); 95% CI for %**					
	Seeing a gastroenterologist	—^a^	—	51 (49.5); 39-59	31 (30.1); 21-40
	Seeing a urologist	—	—	9 (8.7); 4-16	13 (12.6); 7-21
	Accompanying a patient	—	—	43 (41.7); 32-52	59 (57.2); 47-67

^a^A question about the reason for the visit was not asked in study 1.

**Table 2:** Preferred form related to the order of presentation.

Preference	Study 1, n (%); 95% CI for %		Study 2, n (%); 95% CI for %	
	Revised form first (N=86)	Current form first (N=92)	Revised form first (N=103)	Current form first (N=103)
Prefer revised	61 (71); 60-80	50 (54); 44-65	48 (46.6); 37-57	56 (54.4); 44-64
Prefer current	17 (20); 12-30	33 (36); 26-47	35 (34.0); 25-44	29 (28.2); 20-38
Not sure	7 (9); 4-18	8 (9); 4-16	20 (19.4); 12-28	19 (18.5); 12-27

**Table 3:** Preferred form related to having previously undergone a colonoscopy.

Preference	Study 1, n (%); 95% CI for %		Study 2, n (%); 95% CI for %	
	Previous colonoscopy (N=116)	No previous colonoscopy (N=56)	Previous colonoscopy (N=133)	No previous colonoscopy (N=73)
Prefer revised	68 (58.6); 49-68	42 (75); 62-86	69 (51.9); 43-61	34 (46); 35-59
Prefer current	38 (32.8); 24-42	10 (18); 9-30	39 (29.3); 22-38	26 (36); 25-48
Not sure	9 (7.8); 4-14	4 (7); 2-17	25 (18.8); 13-27	14 (19); 11-30

**Table 4:** Predictors of preference for the Revised form.

Predictor	Study 1 (N=154), odds ratio (95% CI)	Study 2 (N=206), odds ratio (95% CI)
Order (0=Current form first, 1=Revised form first)	*3.49 (1.61-7.78)^a^*	0.707 (0.37-1.36)
Previous colonoscopy (0=yes, 1=no)	*2.69 (1.16-6.42)^a^*	1.42 (0.72-2.81)
Gender (0=male, 1=female)	1.76 (0.81-3.74)	1.63 (0.84-3.14)
Age^b^ (0=44 years old or younger, 1=older than 44 years)	1.79 (0.85-3.93)	0.706 (0.36-1.39)
Education sum (0=older than 14 years, 1=14 years old or younger)	1.09 (0.50-2.26)	1.90 (0.99-3.64)
Language spoken at home (0=not English, 1=English)	1.67 (0.39-7.05)	1.82 (0.49-6.79)

^a^Italicized values indicate that the CIs between groups do not overlap.

^b^In study 2, the median split for age used in regression was 0=58 years old or younger, 1=older than 58 years.

Table 4 examines the predictors of preference for the Revised form. In considering the 6 predictors, 2 were significant in study 1. Participants in study 1 were almost 3.5 times as likely to prefer the Revised form if they viewed the Revised form first, a clear order effect. No previous experience with colonoscopy was also associated with a higher preference for the Revised form. In Study 2, none of the 6 variables assessed were associated with preference for the Revised form.

**Figure 1:**
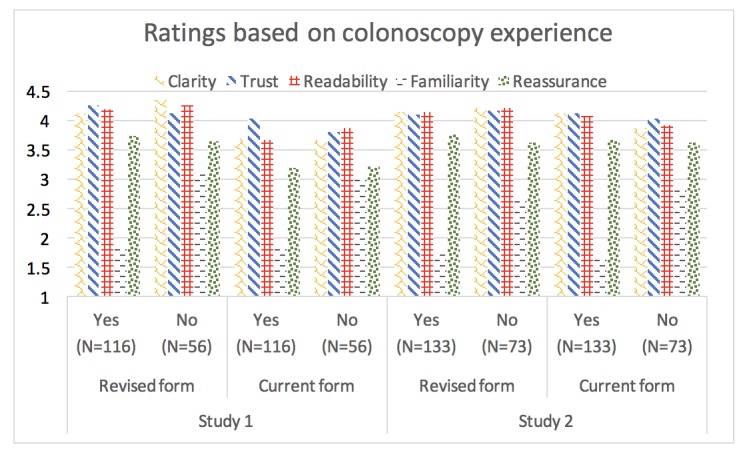
Evaluation of characteristics of the Current and Revised form depending on colonoscopy experience. Yes=previous colonoscopy; No=no previous colonoscopy. Clarity, Trust (=trustworthiness), and readability (=readability/understandability) variables were rated on scales from 1 (strongly disagree) to 5 (strongly agree). Familiarity (=familiarity) variable was rated on a scale from 1 (very familiar) to 5 (very new). Reassurance (=Reassurance) was rated on a scale from 1 (very worried) to 5 (very reassured).

In considering the evaluation of the 2 information forms by colonoscopy experience (Figure 1), the overall pattern of responses in each study was similar. The Revised form in study 1 was given significantly higher ratings of clarity, readability and understandability, and reassurance by participants than the Current form. Not surprisingly, individuals who had previously undergone a colonoscopy indicated that information in both forms was more familiar than those who had not previously undergone a colonoscopy (see Multimedia Appendix 6 for means with 95% CI). In study 2, ratings were more similar between forms, regardless of the colonoscopy experience.

**Figure 2:**
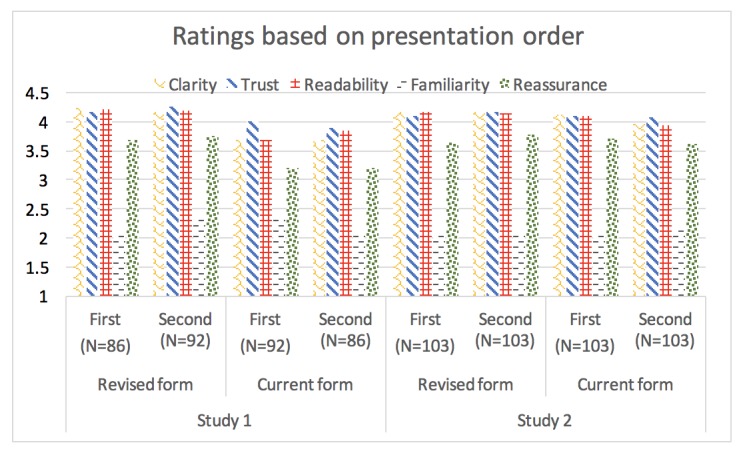
Evaluation of characteristics of the Current and Revised form depending on the order of presentation. Clarity, trust (=trustworthiness), and readability (=readability/understandability) variables were rated on scales from 1 (strongly disagree) to 5 (strongly agree). Familiarity (=familiarity) variable was rated on a scale from 1 (very familiar) to 5 (very new). Reassurance (=Reassurance) was rated on a scale from 1 (very worried) to 5 (very reassured).

In considering the evaluation of the 2 information forms by the order of presentation (Figure 2), the Revised form was rated as significantly clearer, easier to read or understand, and more reassuring than the Current form, regardless of the order it was viewed in study 1. Furthermore, those who viewed the Revised form second in study 1 rated it as significantly more trustworthy than the group that viewed the Current form second (see Multimedia Appendix 7 for means with 95% CI). In Study 2, ratings were more similar between forms, regardless of the order.

In comparing the 2 forms on amount of information (Table 5), most respondents (more than 80%) in Study 1 and Study 2 indicated that the Revised form had just the right amount of information, which was higher than the ratings of just the right amount of information for the Current form. The layout of the educational material using short paragraphs and subheadings allow the reader who is not interested in a topic to skip that topic. They also allow readers to find topics that are important to them. Respondents to the survey, on the other hand, were asked to read all topics of both sets of educational materials—creating a situation where information could have seemed like too much. The proportion of respondents finding information either too little or too much was modest in most cases.

**Table 5:** Proportion of ratings of the amount of information in the educational resource in Study 1 and Study 2. Amount variable was rated on a scale from 1 (*much too little*), 2 (too little), 3 (*just right*) 4 (*too much*), to 5 (*way too much*).

Amount	Study 1 (N=178), n (%); 95% CI for %		Study 2 (N=206), n (%); 95% CI for %	
	Revised form	Current form	Revised form	Current form
Much too little	0	0	1 (0.5); 0-3	1 (0.5); 0-3
Too little	2 (1.1); 0.01-4	55 (30.9); 24-38	6 (3.4); 1-6	17 (8.3); 5-13
Just right	142 (79.8); 73-85	110 (61.8); 54-69	179 (86.9); 82-91	181 (87.9); 83-92
Too much	25 (14.0); 9-20	9 (5.1); 2-9	19 (9.2); 5-14	4 (1.9); 0.5-5
Way too much	7 (3.9); 2-8	0	1 (0.5); 0-3	2 (1.0); 0.1-4

**Figure 3:**
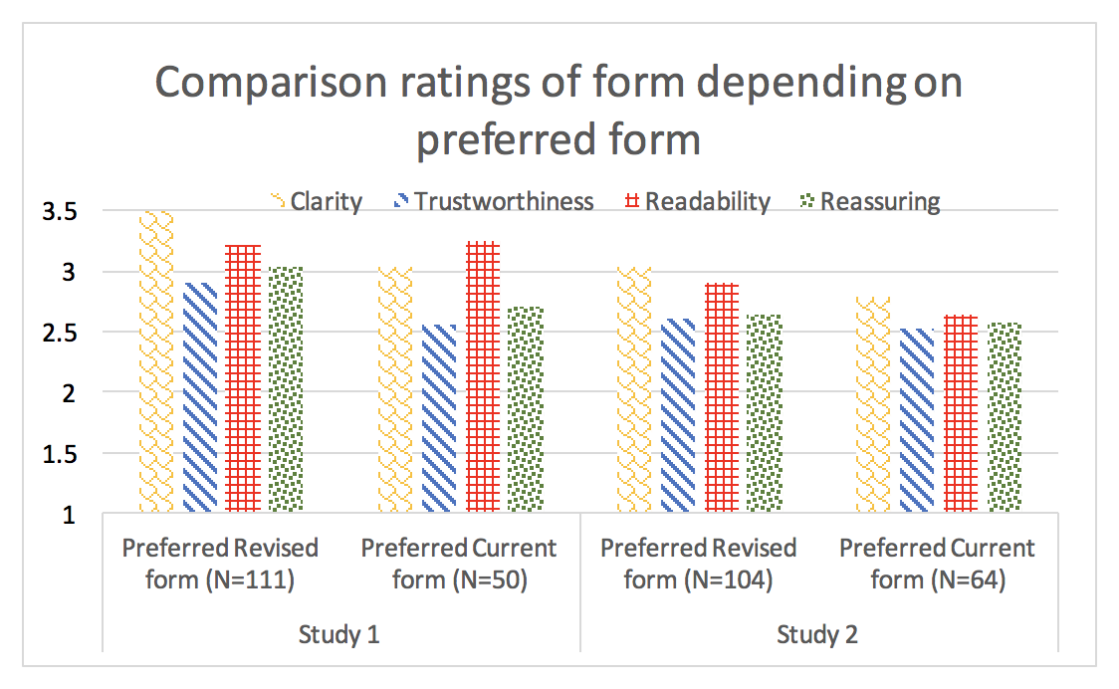
Evaluation comparison ratings of form depending on participants’ preferred form. Rating scale for clarity: 1 (less clear than the form I did not prefer), 2 (about as clear as the form I did not prefer), 3 (somewhat clearer than the form I did not prefer), and 4 (much more clear than the form I did not prefer). Rating scale for trustworthiness: 1 (less trustworthy than the form I did not prefer) to 4 (much more trustworthy than the form I did not prefer). Rating scale for readability: 1 (less easy to read and understand than the form I did not prefer) to 4 (much easier to read and understand than the form I did not prefer). Rating scale for reassuring: 1 (more worrying than the form I did not prefer) to 4 (much more reassuring than the form I did not prefer).

In comparing the 2 forms head-to-head (Figure 3), participants who preferred the Revised form in study 1 rated it as significantly clearer compared with those who preferred the Current form (see Multimedia Appendix 8 for means with 95% CI).

Multimedia Appendix 9 examines the Pearson correlations of the variables used to evaluate the 2 forms. Cohen [29] discusses suggested cut-off scores for small (*r*=.1), medium (*r*=.3), and large (*r*=.5) Pearson correlations. For the Revised form, there were moderate and significant correlations for clarity and trustworthiness, readability and understandability, and reassurance. In addition, moderate and significant correlations for trustworthiness and readability and understandability and trustworthiness and reassurance were found. Finally, there was a moderate and significant correlation between readability and understandability and reassurance. A very similar pattern was observed for ratings of the Current form. The size of the correlations suggests that the concepts are related but not completely overlapping.

In the content analysis of open-ended questions, we found the following in study 1:

- 41 participants liked the length of the Current form because it was shorter than the Revised form.
- An area of confusion was having instructions for more than 1 type of bowel preparation on the information sheets.
- Terminology was problematic for some participants including words such as “endoscopist” and phrases such as “if unable to tolerate the split bowel prep.”
- Many participants commented on the importance of having a section on problem solving and suggestions that includes common issues and concerns with bowel preparation; this was identified by several as a strength of the Revised form.
- Some respondents noted that referring people to a website for more information would be problematic for some people who do not use the internet.
- Others felt that connection with a person to go through the information would be helpful for some patients.
- Many of those who preferred the Revised form indicated that they liked it because of the following features:
  - More information than the Current form;
  - Good visuals;
  - Good layout was important;
  - Colors and highlighting of particular points was helpful;
  - Better spacing and lines between sections were important for a few of the respondents;
  - Slick and professional look; and
  - Clarity of instructions.

The qualitative data obtained in study 2 tell a slightly different story. The following was observed in study 2:

- There was not as strong a preference for the Revised form compared with the Current form.
- Many participants liked the clarity of the Revised form and found it easy to read and understand.
- Many participants liked the shorter length of the Current form and also found it clear and easy to read or understand.
- Some participants indicated that the more information contained in the Revised form was the reason they preferred it.
- Some participants wanted more information about risks and complications, whereas others found the discussion of risks to be “scary.”
- Some participants also found the visuals in the Current form to be a strength, particularly for those who preferred the Current form.
- Format and language of both forms were also found to be important to participants in study 2.

## Discussion

### Principal Findings

This study is one of the first studies to evaluate revised educational materials in a head-to-head comparison with existing materials using the same participants (a within-subjects design). This is significant, as it involves developing a novel approach to comparing consumers’ judgments concerning information quality of different sources of information. This study will contribute to the methodology for evaluating the quality of newly developed information in comparison with existing sources of information.

A recent study by our research group found that patients do not feel adequately informed about the colonoscopy procedure [10]. One way to address this is to provide high-quality educational material to patients before colonoscopy. The 2 main advantages of written educational materials are that they have a low cost (particularly when available on the Web), compared with a telephone or in-person consultation, and patients can review the specific sections of information that are of interest to them. In regular use of health information, consumers may only read the information that is of interest to them (eg, on a specific treatment). The responses from participants in this research will assist in developing improved educational material focused on the information needs of patients.

Another important contribution of this study was the evaluation of order effects. We examined how strongly people are influenced by order effects (ie, primacy or recency effects) when evaluating information. There has been little previous research done in this area, particularly around health information. In study 1, the order of the 2 forms influenced ratings; viewing the Revised form first predicted preference for the Revised form. However, the order did not predict preference for the Revised form in study 2. This is in contrast to the results of a recent study that investigated the order of the presentation of psychological symptom information [30]. The authors found that the order of symptom description predicted the correctness of diagnostic decisions by clinicians; correct diagnostic decisions occurred more often when the symptom information was presented last (ie, a recency effect) [30]. In this study, memory was not an outcome of interest, as participants had access to both forms when making their ratings, and hence, recency did not have a positive effect on ratings. Our study results confirm our hypothesis that counterbalancing is essential in within-subject designs to assess possible order effects, which can vary depending on the material assessed.

Furthermore, ratings of the 2 forms were more similar in study 2 than in study 1. In considering the reason for this, we examined the responses to the open-ended questions; participants especially liked the visual aids, the step-by-step instructions, and the problem-solving section in the study 1 Revised form. Although the Revised form in study 2 was broken down into short sections, there was less use of graphics and the procedure was not considered in a step-by-step process. Moving forward, when developing high-quality information on health procedures, laying out information in a format that the general public is most receptive to is essential.

Another important issue that this study aimed to address is the issue of health literacy. Health literacy is defined as “the personal and relational factors that affect a person’s ability to acquire, understand, and use information about health and health services” [31]. Hence, information materials on a given health issue need to be clearly understandable by persons of varying backgrounds. Previous research has found that health literacy is related to comprehension of colonoscopy information and illustrates the importance of developing materials for people with various levels of health literacy [32].

Regardless of experience, colonoscopy is a procedure that can cause patients considerable anxiety. Some may feel worried about the discomfort of using the bowel preparation laxative, whereas others worry about the outcome of the procedure. Providing quality education materials to patients about colonoscopy (especially for those undergoing a colonoscopy for the first time) is an important step to assist in reducing this anxiety. This study found that the Revised form helped readers to feel more reassured compared with the Current form, which is important in alleviating some of this anxiety. Moreover, the Revised form was also more reassuring for individuals who had never previously undergone a colonoscopy. This is a crucial finding, as no previous colonoscopy experience is typically associated with the most anxiety [33].

### Limitations

This study had a few limitations. The first is that participants were enrolled in this study through waiting room recruitment. Therefore, the number of participants who responded was a factor of how busy the waiting room was at the time and whether they could complete the survey before their appointment. However, the response rate was still reasonable (79%). Some participants were called in to see their physician before they had an opportunity to complete the survey. The survey also included a reasonable number of people (25% to 33%) with no previous experience with colonoscopy. The survey included mainly older adults and had a limited number of people who were younger, who did not use English at home, and who had very limited education. This may limit the generalizability of the findings to these other groups. The experiences in specific stratified sociodemographic groups such as young adults, persons with less education, differing cultural backgrounds, or those with less experience with colonoscopy need additional study. Finally, the information materials were presented in a paper format, and this information is available on our research team’s website. Whether the experience will be different when viewed only on the Web is not known. However, the focus of this study was on the content and layout of the material, as opposed to the usability or navigation ability of the website. Usability is something that should be further studied with the Web version of these information materials.

### Conclusions

Our survey approach with the counterbalanced presentation of information provides a useful approach in comparing revised educational information with resources that are already used in the area and/or alternate revised educational information materials. Moving forward, this knowledge translation approach of a head-to-head comparison of 2 different information sources could be taken to develop and refine information sources on other health issues.

## Appendix

Multimedia Appendix 1 Survey questions.

Multimedia Appendix 2 Colonoscopy bowel preparation instructions Revised form.

Multimedia Appendix 3 Colonoscopy bowel preparation instructions Current form.

Multimedia Appendix 4 About colonoscopy Revised form.

Multimedia Appendix 5 About colonoscopy Current form.

Multimedia Appendix 6 Evaluation of the characteristics of Current and Revised form depending on colonoscopy experience.

Multimedia Appendix 7 Evaluation of the characteristics of Current and Revised form depending on the order of presentation.

Multimedia Appendix 8 Comparison ratings of the forms depending on the preferred form.

Multimedia Appendix 9 Correlations among evaluation variables.

## Abbreviations

RCT: randomized controlled trial
SMOG: Simple Measure of Gobbledygook
