# Supplementary material for: Evaluating Information Quality of Revised Patient Education Information on Colonoscopy: It Is New But Is It Improved?
Source: Interact J Med Res. 2019 Feb 20;8(1):e11938. doi: 10.2196/11938 (PMC6401670; doi:10.2196/11938)
Supplement: Multimedia Appendix 1 [file ijmr_v8i1e11938_app1.pdf]

PATIENT INFORMATION SURVEY

[BP form uv]

Colonoscopy Information

Bowel Preparation Instructions

Our team is evaluating educational materials developed for the public about common medical procedures and decisions. We would appreciate your opinion about two different versions of some of this material. We are interested in which version you think may be more helpful for people considering colonoscopy.

COLONOSCOPY: When a patient sees a doctor specialized in gastroenterology or a family doctor, it is sometimes recommended that they have this test. A colonoscopy is a procedure which allows the inside of the colon (also called large intestine or large bowel) to be examined using a long thin flexible tube with a tiny video camera at the tip. Colonoscopies help doctors diagnose probable causes of rectal bleeding, diarrhea and sometimes, chronic abdominal pain. Colonoscopy is used to look for colon polyps and early signs of colon cancer.

Please carefully read the information sheet labelled FORM U

Your opinion: Considering a person who was going to have a colonoscopy and would like information about this aspect of colonoscopy.

U1. **The amount of information on this topic was:**

( ) much too little

( ) too little

( ) just right

( ) too much

( ) way too much

U2. **The information on this topic is clear.**

( ) strongly disagree

( ) disagree

( ) neutral

( ) agree

( ) strongly agree

U3. **The information on this topic seems trustworthy.**

( ) strongly disagree

( ) disagree

( ) neutral

( ) agree

( ) strongly agree

U4. **The information on this topic is easy to read and understand.**

( ) strongly disagree

( ) disagree

( ) neutral

( ) agree

( ) strongly agree

U5. **How familiar to you or new to you is the information on this topic?**

( ) very familiar

( ) familiar

( ) unsure

( ) new

( ) very new

U6. **This information would leave someone who will be having a colonoscopy feeling**

O very worried

O worried

O neutral

O reassured

O very reassured

U7. **What did you like about the material?**

U8. **What did you dislike about the material?**

U9. **Do you have any suggestions for improving the material or for other things that should be included?**

Please carefully review the information sheet labelled FORM V

Your opinion: Considering a person who was going to have a colonoscopy and would like information about this aspect of colonoscopy.

V1. **The amount of information on this topic was:**

( ) much too little

( ) too little

( ) just right

( ) too much

( ) way too much

V2. **The information on this topic seems clear.**

( ) strongly disagree

( ) disagree

( ) neutral

( ) agree

( ) strongly agree

V3. **The information on this topic seems trustworthy.**

( ) strongly disagree

( ) disagree

( ) neutral

( ) agree

( ) strongly agree

V4. **The information on this topic is easy to read and understand**

( ) strongly disagree

( ) disagree

( ) neutral

( ) agree

( ) strongly agree

V5. **How familiar to you or new to you is the information on this topic?**

( ) very familiar

( ) familiar

( ) unsure

( ) new

( ) very new

V6. **This information would leave someone who will be having a colonoscopy feeling**

O very worried

O worried

O neutral

O reassured

O very reassured

V7. **What did you like about the material?**

V8. **What did you dislike about the material?**

V9. **Do you have any suggestions for improving the material or for other things that should be included?**

Comparing Form U and Form V:

S1. **Which form do you think would be most helpful for people who are considering having a colonoscopy?**

O Form U [GO TO QUESTION S2.]

O Form V [GO TO QUESTION S2.]

O Not sure [SKIP TO THE BACKGROUND QUESTIONS SECTION BELOW, QUESTION S7]

S2. **Comparing your preferred form with the other form, the preferred form is:**

O less **clear** than the form I did not prefer

O about as **clear** as the form I did not prefer

O somewhat more **clear** than the form I did not prefer

O much more **clear** than the form I did not prefer

S3. **Comparing your preferred form with the other form, the preferred form is:**

O less **trustworthy** than the form I did not prefer

O is about as **trustworthy** as the form I did not prefer

O is a somewhat more **trustworthy** than the form I did not prefer

O is much more **trustworthy** than the form I did not prefer

S4. **Comparing your preferred form with the other form, the preferred form is:**

O less easy **to read and understand** than the form I did not prefer

O about the same **to read and understand** as the form I did not prefer

O somewhat easier **to read and understand** than the form I did not prefer

O much easier **to read and understand** than the form I did not prefer

S5. **Comparing your preferred form with the other form, the preferred form is:**

O more **worrying** than the form I did not prefer

O about the **same in reassurance** as the form I did not prefer

O somewhat more **reassuring** than the form I did not prefer

O much more **reassuring** than the form I did not prefer

S6. **Why do you think your preferred form is better?**

Background questions.

This section asks a few questions about you.

S7. **Have you seen a specialist in gastroenterology before?**

O No

O Yes If yes about how many times: ______

S8. **Have you ever had a colonoscopy?**

O No

O Yes **If yes about how many times**: ______

S9. **IF YES, when did you have the last colonoscopy?**

O Within the past year

O One to two years ago

O More than 2 years but less or equal to 5 years ago

O More than 5 years but less or equal to 10 years ago

O More than 10 years ago

S10. **Your sex:** O Male O Female

S12. **Your age:** ______

How many years of education have you completed in the following areas?

S13. **Completed high school diploma:** O Yes O No

S14. **Apprenticeship:** O 0 O 1 O 2 O 3 O 4 O 5 years

S15. **College, technical, business, vocational, nursing (non-university):**

O 0 O 1 O 2 O 3 O 4 O 5 O 6 years

S16. **University program**:

O 0 O 1 O 2 O 3 O 4 O 5 O 6 O 7 O 8 O 9 O 10 O 11 O 12 or more

S17. **What is the language you most often speak at home?**

O English

O French

O Another language **What language?** ________________

S18. **Your postal code:** __ __ __ __ __ __

Thank you for completing the survey. The information you provide will help us improve the information available to people when they are preparing for a colonoscopy!
