# Supplementary material for: Evaluating Information Quality of Revised Patient Education Information on Colonoscopy: It Is New But Is It Improved?
Source: Interact J Med Res. 2019 Feb 20;8(1):e11938. doi: 10.2196/11938 (PMC6401670; doi:10.2196/11938)
Supplement: Multimedia Appendix 2 [file ijmr_v8i1e11938_app2.pdf]

INFORMED CHOICES ABOUT

COLONOSCOPY

Bowel Prep Instructions

Very important!

Follow these instructions to clean the stool (poop) out of your colon. Carefully following these instructions will give the best results. If the doctor cannot see the inside of your colon well because of poor cleaning, the test is less useful and very important things can be missed. You may have to repeat it another time. The risks of the test are more if the colon is not clean.

!

Your colonoscopy doctor may give you instructions for preparation that are different. Please follow those if they are different than below.

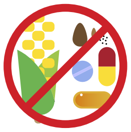

Step 1. One week before your colonoscopy: Do not eat corn, flax/poppy/sesame seeds, nuts, fish oils, or medicines containing iron including multivitamins containing iron.

Step 2. A few days before your colonoscopy: Go to any pharmacy and buy the following special laxatives. You do not need a prescription but they may be kept behind the pharmacy counter. Ask the pharmacist. (Your doctor may suggest different laxatives than the ones below. Please follow those instructions.)

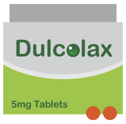

- 2 Dulcolax (also called bisacodyl) 5 mg tablets

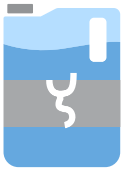

- 4 litre jug of Lyte prep (Golytely or Colyte or PegLyte or Klean-Prep)–this is powder in a jug that is later mixed with water. This is most commonly recommended.
- Your doctor may ask you to use another laxative such as Pico-Salax or Bi-PegLyte as alternatives to the "Lyte" medicines above. These require a lower amount of liquid. Use the laxative your doctor recommends

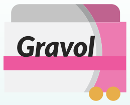

- Some people have a problem with nausea when taking the laxatives. You may want to pick up an anti-nausea medicine like Gravol. The pharmacist can show you the choices for this.

©2017 Harminder Singh, John R. Walker on behalf of Manitoba Optimizing Colonoscopy Project. This work is licensed under a Creative Commons Attribution-nonCommercial-NoDerivs 2.5 Canada License. You are free to copy and distribute this material in its entirety as long as: 1) this material is not used in any way that suggests we endorse you or your use of the material, 2) this material is not used for commercial purposes, 3) this material is not altered in any way (no derivative works). View full license at http://creativecommons.org/licenses/by-nc-nd/2.5/ca/.

INFORMED CHOICES ABOUT

COLONOSCOPY

Step 3. The day before your colonoscopy

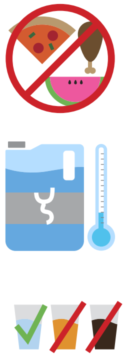

- Do not eat any solid foods–until the colonoscopy is finished.
- Drink only clear fluids–until two hours before the time of the colonoscopy. These are liquids that you can see through. See the ‘Fluids that are okay’ list below. Coffee or tea are okay with sweetener but no milk or whitener.
- Try to drink one glass of clear fluids each hour that you are awake.
- Mix the liquid laxative according to the instructions. You may want to keep it in the refrigerator so that it is cold. Many people find that it tastes better if it is cold.

Fluids that are okay:	Food and Fluids that are not okay:
✓ clear soups	✗ no bread, grain or rice
✓ apple juice, **white** grape juice, **white** cranberry juice	✗ no soups with chunks of food
✓ clear of lemon Gatorade/Powerade	✗ no meat
✓ ginger ale, and water	✗ no fruit or vegetables
✓ coffee or tea (sugar and sweetner are okay)	✗ no milk or dairy products
✓ popsicles (not red or purple)	✗ no red, blue or purple liquid
✓ lemon or orange Jello	✗ no alcholic drinks

Step 4. The day before the colonoscopy at 6:00 PM–taking the laxatives

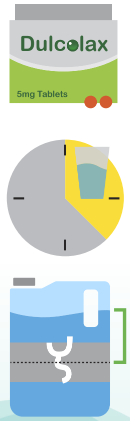

- Take the 2 Dulcolax (bisacodyl) tablets
- Start drinking the liquid laxative (250 ml (1 cup) of Lyte prep) every 10 to 15 minutes until you finish half the container (2 litres or 8 cups). Drink the first half of the 4 litre container within 2 hours (by 8 pm). If your doctor recommended a different laxative take the first half of that laxative between 6 and 8 pm.
- If you feel sick, wait 20 to 30 minutes, then continue to drink the prep.

Mobilizing Minds Research Group © 2017 John R. Walker and John D. Eastwood.
This work is licensed under a Creative Commons Attribution-NonCommercial-NoDerivs 2.5 Canada License.
To view a copy of this license, visit http://creativecommons.org/licenses/by-nc-nd/2.5/ca/.

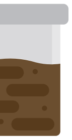

- You may take Gravol (25 to 50 mg) or a similar medicine if the nausea continues. Gravol may make you drowsy.

- It is important to take all of the laxatives. The laxatives will cause frequent loose bowel movements. The goal is to have liquid bowel movements, clear to yellow in colour. **(Remember, when you drive through a snow storm it is difficult to see the road. Similarly, your doctor can't get a clear view for the colonoscopy unless your colon is very clean.)**

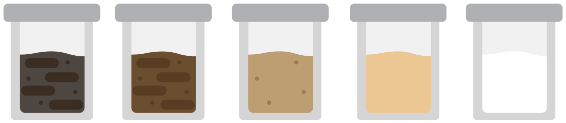

Step 5. The day of your colonoscopy

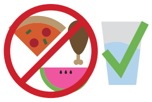

- Eat no solid foods, drink only clear fluids

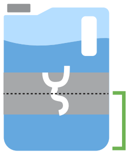

- 4-5 hours before your colonoscopy time, drink 250 ml (1 cup) of Lyte prep every 10 to 15 minutes until you finish the second half of the container (2 litres or 8 cups). Finish the container within 1-2 hours. If your doctor recommended a different laxative, take the second half of it 4 hours before your colonoscopy.

- Stop drinking all fluids at least 2 hours before your colonoscopy

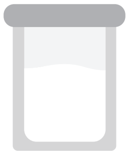

- Your bowel movements should be liquid, clear to yellow in colour which allows your doctor to see your bowel more clearly.

Mobilizing Minds Research Group © 2017 John R. Walker and John D. Eastwood.
This work is licensed under a Creative Commons Attribution-NonCommercial-NoDerivs 2.5 Canada License.
To view a copy of this license, visit http://creativecommons.org/licenses/by-nc-nd/2.5/ca/.

INFORMED CHOICES ABOUT

COLONOSCOPY

Step 6. Have someone take you to the test and someone to take you home from the test

- It is important to have someone with you who can stay to hear the results provided after the test and then take you home. Ask them write down the results and whether another colonoscopy is suggested in future years. You will feel drowsy after the colonoscopy due to the medications used to relax you. You may not remember things you are told about the results. This means it would be dangerous to drive a car immediately after the test.

- Someone should be with you for up to 24 hours afterwards while the drowsiness and forgetfulness wears off. You will not be able to drive a car.

Questions about your colonoscopy: If you have questions about the preparation for the colonoscopy, or if you run into problems, you are welcome to call the office of the doctor who will do the colonoscopy (unless you were given instructions to call a different number). The staff there are used to handling questions. Most of the offices are open on regular business days from 9 AM to 4 PM. Your pharmacist will be very familiar with your regular medications and the different laxatives used for preparing for the colonoscopy and can also provide helpful information.

Cancellation: If you have to cancel the test please inform the colonoscopy booking office as soon as possible. Please remember that if you cancel at the last minute or miss the appointment without cancelling, that will lead to wasted time when a colonoscopy could have been done for someone else who is waiting. If you miss or cancel your colonoscopy, there may be a several months wait for a rebooked colonoscopy. Hence it is important to call as soon as possible.

Results of your colonoscopy: In addition to the results you receive just after your colonoscopy you may receive a written report. If you have questions about the results contact the doctor who did the colonoscopy or your family doctor.

Mobilizing Minds Research Group © 2017 John R. Walker and John D. Eastwood.
This work is licensed under a Creative Commons Attribution-NonCommercial-NoDerivs 2.5 Canada License.
To view a copy of this license, visit http://creativecommons.org/licenses/by-nc-nd/2.5/ca/.

INFORMED CHOICES ABOUT

COLONOSCOPY

Problem Solving

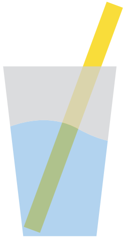

Problem: You may find it hard to drink all of the liquid. Some people don't like the taste and some have difficulty in drinking the amount needed to clean the colon properly.

Suggestion: Some people find that drinking with a straw helps – so you don't taste it as much. If the powder is not flavored you can add flavor crystals. You can rinse mouth out with a clear drink after each glassful (e.g. ginger ale, 7 Up®, apple juice). If you feel really full, it can help to walk around.

- Cool it to your preferred temperature in the fridge. If you start to feel cold you can take it out of the fridge or drink some clear warm liquid like broth or herbal tea.

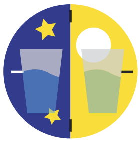

Problem: If your colonoscopy is scheduled for a morning time (say 9 AM) you will have to get up at 4 or 5 AM. Some people find this is difficult.

Suggestion: Research shows that taking half of the liquid laxative the evening before the test and half the morning of the test is better for cleaning the bowel and results in better colonoscopies. Remember that people often have to get up early before a long trip also. Plan ahead for some activities you can do early in the morning (radio, music, TV, computer) while taking frequent trips to the toilet.

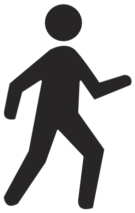

Problem: Some people feel gassy after the test because of the air used to help do the colonoscopy.

Suggestion: Walking may help with this feeling.

Problem: You have a long drive to reach the colonoscopy facility/hospital.

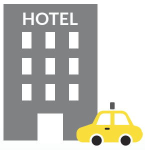

Suggestion: Most people find that they do not have to make frequent visits to the toilet in the two hours before the colonoscopy. If you have to travel for more than one hour, it may be best to stay somewhere closer to the colonoscopy facility starting the evening before the colonoscopy. You should try to finish your laxative in the morning half to one hour before you leave your home/hotel.

Mobilizing Minds Research Group © 2017 John R. Walker and John D. Eastwood.
This work is licensed under a Creative Commons Attribution-NonCommercial-NoDerivs 2.5 Canada License.
To view a copy of this license, visit http://creativecommons.org/licenses/by-nc-nd/2.5/ca/.
