# Supplementary material for: Evaluating Information Quality of Revised Patient Education Information on Colonoscopy: It Is New But Is It Improved?
Source: Interact J Med Res. 2019 Feb 20;8(1):e11938. doi: 10.2196/11938 (PMC6401670; doi:10.2196/11938)
Supplement: Multimedia Appendix 3 [file ijmr_v8i1e11938_app3.pdf]

Bowel Prep Instructions

Very important! Follow these instructions to clean the stool (poop) out of your colon. If the doctor cannot see the inside of your colon on the day of your colonoscopy, you may have to repeat the procedure. Your endoscopist may provide you with alternative instruction for preparation. Please follow those if they are different than below.

******If you have any questions regarding this bowel prep please call your endoscopist's office**

Preparing for your colonoscopy:

For 1 week before your colonoscopy do not eat flax/poppy/sesame seeds or fish oils. A few days before your colonoscopy, go to any pharmacy and buy:

- 4 litre jug of Lyte prep (Golytely or Colyte)
- 2 Dulcolax (Bisacodyl) 5 mg tablets

You do not need a prescription for these medications, but they may be kept behind the counter. Ask the pharmacist

The day before your colonoscopy:

Do not eat any solid foods.

- Drink only clear fluids. These are liquids that you can see through. See the 'Fluids that are okay' list on the back of this page.
- Try to drink one glass of clear fluids each hour that you are awake.
- Take your bowel preparation as described below:

SPLIT BOWEL PREP INSTRUCTIONS:

Use this bowel prep for morning and afternoon scopes – this is the best way to prepare for your colonoscopy

- Mix your Lyte prep as described on the jug.
- Beginning at 6:00 p.m., take the 2 Dulcolax tablets and start drinking 250 ml (1 cup) of Lyte prep every 10 to 15 minutes until you finish half the jug (2 litres or 8 cups). You must drink the first half of the jug within 2 hours (by 8 pm).
- If you feel sick, wait 20 to 30 minutes, then continue to drink the prep.
- You may take Gravol (25 to 50 mg) if the nausea continues. Gravol may make you drowsy.
- The prep will cause frequent loose bowel movements. The goal is to have liquid bowel movements, clear to yellow in colour.

The day of your colonoscopy:

- Do not eat any solid foods.
- Drink only clear fluids. It is important to stop drinking all clear fluids 3 hours before your colonoscopy
- 5 hours before your procedure drink 250 ml (1 cup) of Lyte prep every 10 to 15 minutes until you finish the second half of the jug (2 litres or 8 cups). You must finish the jug within 2 hours.

NON-SPLIT BOWEL PREP INSTRUCTIONS:

Use this bowel prep for scopes in morning only if unable to tolerate the split bowel prep

- Mix your Lyte prep as described on the jug.
- Beginning at 6:00 p.m., take the 2 Dulcolax tablets and start drinking 250 ml (1 cup) of Lyte prep every 10 to 15 minutes until you finish the full jug (4 litres or 16 cups). You must drink the full jug within 4 hours (by 10 pm).
- If you feel sick, wait 20 to 30 minutes, then continue to drink the prep.
- You may take Gravol (25 to 50 mg) if the nausea continues. Gravol may make you drowsy.
- The prep will cause frequent loose bowel movements. The goal is to have liquid bowel movements, clear to yellow in colour.

The day of your colonoscopy:

- Do not eat any solid foods.
- Drink only clear fluids. It is important to stop drinking all clear fluids 3 hours before your colonoscopy.

Fluids that are okay:

- clear soup broth or bouillon
- apple juice, **white** grape juice, **white** cranberry juice
- clear or lemon Gatorade / Powerade
- ginger ale, and water
- coffee or tea (sugar and sweetener are okay)
- popsicles (not red or purple)
- lemon or orange Jell-o

Food and fluids that are not okay:

- No bread, grain, or rice
- No soups with chunks of food
- No meat
- No fruit or vegetables
- No milk or dairy products
- No red, blue, or purple liquid
- No alcoholic drinks

You know that your bowel preparation is complete when your stool (poop) is a yellow to clear coloured liquid:

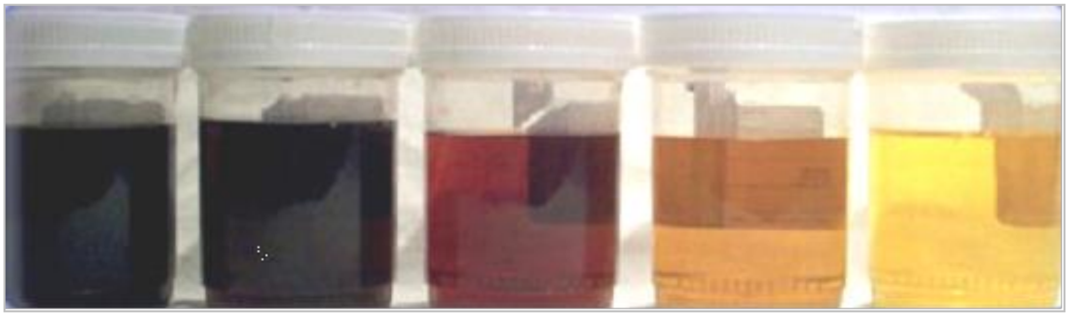

Not okay | **Not okay** | **Not okay** | **Almost there…** | **You are ready!**

For additional information: http://www.wrha.mb.ca/prog/Endoscopy/Procedure-Information.php
