# Supplementary material for: Evaluating Information Quality of Revised Patient Education Information on Colonoscopy: It Is New But Is It Improved?
Source: Interact J Med Res. 2019 Feb 20;8(1):e11938. doi: 10.2196/11938 (PMC6401670; doi:10.2196/11938)
Supplement: Multimedia Appendix 5 [file ijmr_v8i1e11938_app5.pdf]

Winnipeg Regional Health Authority
Caring for Health

Office régional de la santé de Winnipeg
À l'écoute de notre santé

COLONOSCOPY INSTRUCTIONS

Please take the time to read this information. It will help you understand and prepare for your colonoscopy.
Please remember there is a waiting list for these procedures.

******If you have any questions about your bowel prep or the procedure itself (diet, medications), please call your endoscopist's office.**

******If you must reschedule your appt please call Central Intake Endoscopy office at (204) 940-2333**

OVERVIEW: A colonoscopy is a procedure which allows the inside of the colon (large intestine) to be examined using a long thin flexible tube with a tiny video camera at the tip. You will be sedated for this procedure. The procedure lasts approximately 20 to 45 minutes. You will spend another 30 minutes to an hour recovering from the sedation given for the test.

Colonoscopy is the most effective method for diagnosing colon diseases (such as Colitis, Crohn's disease or blockages), and for diagnosing and preventing colon cancer by finding and removing polyps (pre-cancerous growths). Having a colonoscopy has been associated with a decreased risk of developing or dying of colon cancer in the future.

PROCEDURE: When you arrive at the scoping department, you will meet a nurse who will briefly review your medical history. You will change into a hospital gown and have your vital signs measured. An intravenous (IV) line will be placed in your arm. This IV line will be used to provide your sedating drugs (midazolam and fentanyl) when your procedure is started. You will be taken on a stretcher to the procedure room and you will be met by your doctor and the nurses. You will be positioned on your left side. You will be given oxygen, and your blood pressure, heart rate and breathing will be monitored throughout the entire procedure. Once you are sedated, the doctor will start the examination by inserting an examining finger into the rectum. Then the tube, which is the thickness of the examining finger, will be inserted into your anus. Many patients are anxious about the procedure but more than 90% of patients report a comfortable experience. Please express any of your concerns to the nurse or doctor before your procedure. Also, there may be medical students, nursing students or resident physicians present and/or participating in your procedure.

RISKS: Complications are rare. Minor complications, which include medication reactions or difficulty breathing due to the sedatives, temporary bleeding, abdominal pain, discomfort, or bloating, occur in about 1:100 (1%). Major complications, such as perforation or a hole being made in the intestine, are rare and occur in less than 1:1000 (0.1%), and surgery and/or hospitalization may be required to manage it in about 1:3000 (0.03%).

INSTRUCTIONS

BOWEL PREPARATION: The preparation of the bowel prior to colonoscopy is the MOST IMPORTANT part of the procedure that you can control. A good bowel prep allows a quicker examination, better ability to find precancerous and cancerous growths, and can prevent the need for repeat colonoscopies!

PLEASE TAKE THE TIME TO READ THE ATTACHED **BOWEL PREPARATION INSTRUCTIONS**.

MEDICATIONS: Do not interrupt any of your medications during your preparation for this test. You can take all of your medication with sips of clear fluids. If you are on blood thinning medications/anticoagulants (such as Aspirin, Plavix, warfarin, dabigatran, rivaroxaban etc.), please continue them unless instructed otherwise. If your test requires blood thinners to be stopped, the endoscopist performing this procedure will specify this for you.

If you are diabetic on insulin or oral diabetic medications, please follow the attached diabetes management instruction sheets and do not hesitate to call your doctor's office for more information.

OTHER PREP INSTRUCTIONS: Please bring a list of your medications with you to the hospital. Please do not wear nail polish on your fingernails or any jewelry to your test. Please do not bring valuables.

AFTER THE TEST: Your doctor will provide you with a note regarding the test outcome, as well as some post procedural information. Any biopsies or polyps that are removed will be reviewed by the pathologist. Results are available within a month. If there are any concerning or important findings, you will be called directly by your doctor or their nurse, or you may be asked to return to clinic to discuss the results. A letter will also be sent to your referring physician to inform them of the results and outline further treatment plans if necessary.

GOING HOME: **If you have sedation with your procedure:** After the procedure, a person spends about 30 minutes to one hour waking up in the recovery room until the medications wear off. After recovery, you will be discharged from hospital. You cannot drive for **24 hours**, and you require a responsible person to pick you up from the hospital and take you home. It is **required** that you have an adult stay with you the rest of the day and overnight to ensure your safety. **If you have no sedation with your procedure**: You can go home or back to work immediately post procedure. You do not need any supervision and are able to drive yourself.

Drawing of a Colonoscopy:

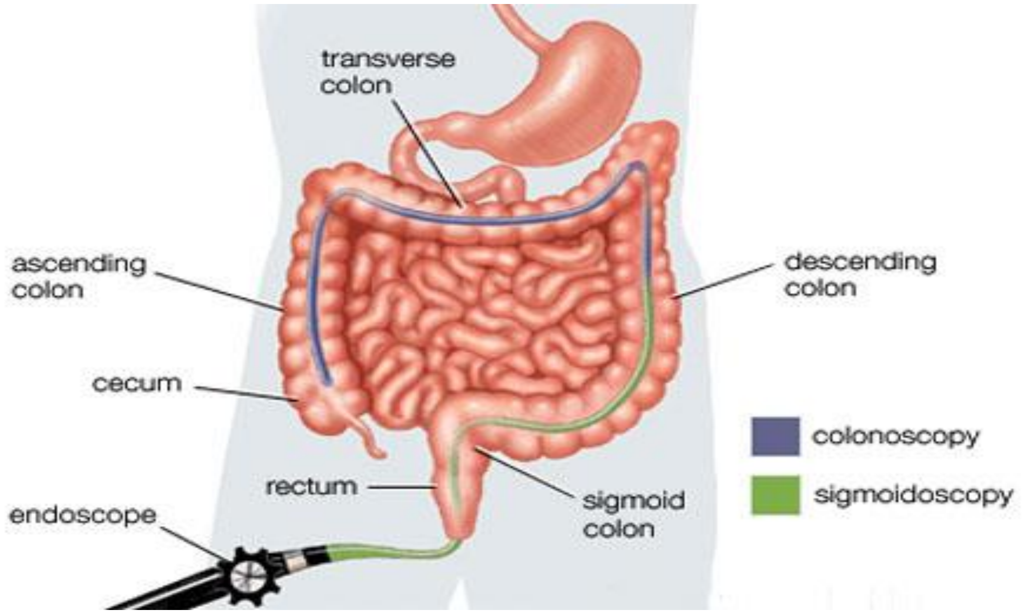

If you have any questions regarding the procedure or issues related to your medications or preparation instructions, please call your doctor's office or the office of the endoscopist performing the scope.

For additional information: http://www.wrha.mb.ca/prog/Endoscopy/Procedure-Information.php
