# Supplementary material for: Evaluating Information Quality of Revised Patient Education Information on Colonoscopy: It Is New But Is It Improved?
Source: Interact J Med Res. 2019 Feb 20;8(1):e11938. doi: 10.2196/11938 (PMC6401670; doi:10.2196/11938)
Supplement: Multimedia Appendix 6 [file ijmr_v8i1e11938_app6.pdf]

Multimedia Appendix 6. Evaluation of Characteristics of Current and Revised Form Depending on Colonoscopy Experience

	Study 1				Study 2			
	Revised form *Mean* (95% CI)		Current form *Mean* (95% CI)		Revised form *Mean* (95% CI)		Current form *Mean* (95% CI)	
	Yes (N=116)	No (N=56)	Yes (N=116)	No (N=56)	Yes (N=133)	No (N=73)	Yes (N=133)	No (N=73)
Clarity (1-5)	*4.13* 4.01,4.26	*4.36* 4.18,4.53	*3.69* 3.53, 3.85	*3.68* 3.46, 3.90	*4.14* 3.98, 4.23	*4.21* 4.08, 4.33	*4.13* 4.04, 4.21	*3.89* 3.73, 4.05
Trust (1-5)	*4.27* 4.16,4.37	*4.13* 3.94,4.31	*4.03* 3.92, 4.14	*3.82* 3.63, 4.01	*4.11* 4.02, 4.25	*4.18* 4.04, 4.31	*4.12* 4.04, 4.20	*4.03* 3.86, 4.20
Read-ability (1-5)	*4.19* 4.05,4.33	*4.27* 4.09,4.44	*3.67* 3.49, 3.85	*3.89* 3.71, 4.07	*4.14* 4.02, 4.25	*4.21* 4.06, 4.35	*4.08* 3.98, 4.18	*3.92* 3.74, 4.10
Familiar (1-5)	*1.83* 1.66,1.99	*3.14* 2.81,3.47	*1.83* 1.68, 1.99	*3.07* 2.74, 3.40	*1.72* 1.58, 1.86	*2.67* 2.40, 2.94	*1.68* 1.55, 1.81	*2.84* 2.54, 3.13
Reassure (1-5)	*3.75* 3.61,3.90	*3.66* 3.47,3.84	*3.20* 3.05, 3.36	*3.21* 3.01, 3.42	*3.76* 3.62, 3.89	*3.64* 3.46, 3.83	*3.68* 3.56, 3.81	*3.63* 3.49, 3.77

Note. Yes = previous colonoscopy; No = no previous colonoscopy. Clarity, Trust (=trustworthiness), and readability (=readability/understandability) variables were rated on scales from 1 (strongly disagree) to 5 (strongly agree). Familiar (=familiarity) variable was rated on a scale from 1 (*very familiar*) to 5 (*very new*). Reassure (=Reassurance) was rated on a scale from 1 (*very worried*) to 5 (*very reassured*).
