# Supplementary material for: Evaluating Information Quality of Revised Patient Education Information on Colonoscopy: It Is New But Is It Improved?
Source: Interact J Med Res. 2019 Feb 20;8(1):e11938. doi: 10.2196/11938 (PMC6401670; doi:10.2196/11938)
Supplement: Multimedia Appendix 7 [file ijmr_v8i1e11938_app7.pdf]

Multimedia Appendix 7. Evaluation of Characteristics of Current and Revised Form Depending on Order of Presentation

	Study 1				Study 2			
	Revised Form *Mean* (95% CI)		Current Form *Mean* (95% CI)		Revised Form *Mean* (95% CI)		Current Form *Mean* (95% CI)	
Order	First (N=86)	Second (N=92)	First (N=92)	Second (N=86)	First (N=103)	Second (N=103)	First (N=103)	Second (N=103)
Clarity (1-5)	*4.25* 4.12, 4.38	*4.18* 4.03, 4.33	*3.69* 3.50, 3.88	*3.69* 3.54, 3.85	*4.16* 4.03, 4.28	*4.18* 4.05, 4.30	*4.12* 4.00, 4.23	*3.97* 3.86, 4.09
Trust (1-5)	*4.18* 4.03, 4.33	*4.27* 4.16, 4.37	*4.01* 3.88, 4.15	*3.89* 3.76, 4.03	*4.11* 3.95, 4.27	*4.16* 4.05, 4.26	*4.10* 3.98, 4.21	*4.08* 3.96, 4.19
Read-ability (1-5)	*4.21* 4.05, 4.38	*4.20* 4.04, 4.36	*3.68* 3.47, 3.89	*3.84* 3.68, 3.99	*4.18* 4.05, 4.32	*4.14* 4.02, 4.26	*4.11* 3.99, 4.22	*3.94* 3.79, 4.09
Familiar (1-5)	*2.11* 1.85, 2.36	*2.38* 2.13, 2.63	*2.37* 2.11, 2.62	*2.08* 1.86, 2.30	*2.07* 1.85, 2.29	*2.07* 1.86, 2.27	*2.06* 1.85, 2.27	*2.13* 1.90, 2.35
Reassure (1-5)	*3.69* 3.52, 3.86	*3.76* 3.60, 3.92	*3.21* 3.05, 3.38	*3.21* 3.03, 3.39	*3.65* 3.51, 3.79	*3.78* 3.61, 3.94	*3.70* 3.57, 3.83	*3.63* 3.50, 3.77

Note. Clarity, Trust (=trustworthiness), and readability (=readability/understandability) variables were rated on scales from 1 (strongly disagree) to 5 (strongly agree). Familiar (=familiarity) variable was rated on a scale from 1 (*very familiar*) to 5 (*very new*). Reassure (=Reassurance) was rated on a scale from 1 (*very worried*) to 5 (*very reassured*).
