# Supplementary material for: Evaluating Information Quality of Revised Patient Education Information on Colonoscopy: It Is New But Is It Improved?
Source: Interact J Med Res. 2019 Feb 20;8(1):e11938. doi: 10.2196/11938 (PMC6401670; doi:10.2196/11938)
Supplement: Multimedia Appendix 8 [file ijmr_v8i1e11938_app8.pdf]

Multimedia Appendix 8. Comparison Ratings of Form Depending on Preferred Form

	Study 1		Study 2	
	Preferred Revised form (N=111)	Preferred Current form (N=50)	Preferred Revised form (N=104)	Preferred Current form (N=64)
1. Clarity compared to other form	3.50 (3.37, 3.62)	3.04 (2.79, 3.29)	3.04 (2.90, 3.18)	2.78 (2.56, 3.00)
2. Trustworthiness compared to other form	2.90 (2.75, 3.05)	2.56 (2.32, 2.82)	2.60 (2.44, 2.75)	2.53 (2.34, 2.73)
3. Readability/ understandability compared to other form	3.22 (3.05, 3.39)	3.24 (3.01, 3.47)	2.91 (2.76, 3.07)	2.64 (2.42, 2.86)
4. Reassuring compared to other form	3.03 (2.88, 3.18)	2.71 (2.45, 2.96)	2.64 (2.50, 2.77)	2.58 (2.40, 2.76)

Note. Rating scale for clarity is: 1 (less clear than the form I did not prefer), 2 (about as clear as the form I did not prefer), 3 (somewhat more clear than the form I did not prefer) to 4 (much more clear than the form I did not prefer). Rating scale for trustworthiness: 1 (less trustworthy than the form I did not prefer) to 4 (much more trustworthy than the form I did not prefer). Rating scale for readability: 1 (less easy to read and understand than the form I did not prefer) to 4 (much easier to read and understand than the form I did not prefer). Rating scale for reassurance: 1 (more worrying than the form I did not prefer) to 4 (much more reassuring than the form I did not prefer).
