# Supplementary material for: Evaluating Information Quality of Revised Patient Education Information on Colonoscopy: It Is New But Is It Improved?
Source: Interact J Med Res. 2019 Feb 20;8(1):e11938. doi: 10.2196/11938 (PMC6401670; doi:10.2196/11938)
Supplement: Multimedia Appendix 9 [file ijmr_v8i1e11938_app9.docx]

**Multimedia Appendix 9**

Correlations Among Evaluation Variables

| Study | Study 1 | | | | | Study 2 | | | | | |
| --- | --- | --- | --- | --- | --- | --- | --- | --- | --- | --- | --- |
| Variable | 1. | 2. | 3. | 4. | 5. | 1. | | 2. | 3. | 4. | 5. |
| Revised form | | | | | | | | | | | |
| 1. Clarity | 1.0 |  |  |  |  | 1.0 | |  |  |  |  |
| 2. Trustworthy | .44 ^a^ | 1.0 |  |  |  | .67 ^a^ | | 1.0 |  |  |  |
| 3. Readability/  understandability | .55 ^a^ | .41 ^a^ | 1.0 |  |  | .63 ^a^ | | .55 ^a^ | 1.0 |  |  |
| 4. Familiarity | -.02 | -.12 | -.02 | 1.0 |  | -.05 | | -.05 | -.06 | 1.0 |  |
| 5. Reassurance | .35 ^a^ | .36 ^a^ | .50 ^a^ | -.02 | 1.0 | .49 ^a^ | | .36 ^a^ | .42 ^a^ | -.03 | 1.0 |
| Current form | | | | | | | | | | | |
| 1. Clarity | 1.0 |  |  |  |  | 1.0 |  | |  |  |  |
| 2. Trustworthy | .50 ^a^ | 1.0 |  |  |  | .63 ^a^ | 1.0 | |  |  |  |
| 3. Readability/  understandability | .52 ^a^ | .23 ^a^ | 1.0 |  |  | .67 ^a^ | .58 ^a^ | | 1.0 |  |  |
| 4. Familiarity | -.05 | -.06 | .12 | 1.0 |  | -.24 ^a^ | -.10 | | -.16 ^b^ | 1.0 |  |
| 5. Reassurance | .29 ^a^ | .25 ^a^ | .31 ^a^ | .13 | 1.0 | .37 ^a^ | .25 ^a^ | | .31 ^a^ | -.08 | 1.0 |

*Note.* ^a^ denotes significance at the .01 level (2-tailed).

^b^ denotes significance at the .05 level (2-tailed).
